# Prevalence and correlations of schistosomiasis mansoni and schistosomiasis haematobium among humans and intermediate snail hosts: a systematic review and meta-analysis

**DOI:** 10.1186/s40249-024-01233-0

**Published:** 2024-09-02

**Authors:** Xin-Yao Wang, Qin Li, Yin-Long Li, Su-Ying Guo, Shi-Zhu Li, Xiao-Nong Zhou, Jia-Gang Guo, Robert Bergquist, Saleh Juma, Jian-Feng Zhang, Kun Yang, Jing Xu

**Affiliations:** 1 National Key Laboratory of Intelligent Tracking and Forecasting for Infectious Diseases, National Institute of Parasitic Diseases at Chinese Center for Disease Control and Prevention (Chinese Center for Tropical Diseases Research), NHC Key Laboratory of Parasite and Vector Biology, WHO Collaborating Centre for Tropical Diseases, National Center for International Research on Tropical Diseases, No. 207 Ruijin 2nd Road, Shanghai, 200025 China; 2 Jiangsu Institute of Parasitic Diseases, Wuxi, 214064 Jiangsu China; 3 Key Laboratory on Technology for Parasitic Disease Prevention and Control, Ministry of Health, Wuxi, 214064 Jiangsu China; 4 Jiangsu Provincial Key Laboratory on the Molecular Biology of Parasites, Wuxi, 214064 Jiangsu China; 5 School of Global Health, Chinese Center for Tropical Diseases Research, Shanghai Jiao Tong University School of Medicine, One Health Center, Shanghai Jiao Tong University-The University of Edinburgh, Shanghai, 200025 China; 6 WHO Department of Control of Neglected Tropical Diseases, Geneva, Switzerland; 7 Geospatial Health, Ingerod, formerly UNICEF/UNDP/World Bank/WHO Special Programme for Research and Training in Tropical Diseases (TDR), Brastad, Sweden; 8 Ministry of Health of Zanzibar, P.O. Box 236, Zanzibar, United Republic of Tanzania; 9 School of Public Health, Nanjing Medical University, Nanjing, China

**Keywords:** *Schistosoma mansoni*, *Schistosoma haematobium*, *Bulinus*, *Biomphalaria*, Prevalence, Correlation analysis, Meta-analysis, Africa

## Abstract

**Background:**

The control of schistosomiasis is particularly difficult in sub-Saharan Africa, which currently harbours 95% of this disease. The target population for preventive chemotherapy (PC) is expanded to all age group at risk of infection, thus increasing the demands of praziquantel (PZQ) tablets according to the new released guideline by World Health Organization. Due to the gap between available PZQ for PC and requirements, alternative approaches to assess endemicity of schistosomiasis in a community, are urgently needed for more quick and precise methods. We aimed to find out to which degree the infection status of snails can be used to guide chemotherapy against schistosomiasis.

**Methods:**

We searched literature published from January 1991 to December 2022, that reported on the prevalence rates of *Schistosoma mansoni*, *S. haematobium* in the intermediate snails *Biomphalaria* spp. and *Bulinus* spp., respectively, and in humans. A random effect model for meta-analyses was used to calculate the pooled prevalence estimate (PPE), with heterogeneity assessed using I-squared statistic (*I*^*2*^), with correlation and regression analysis for the exploration of the relationship between human *S. mansoni* and *S. haematobium* infections and that in their specific intermediate hosts.

**Results:**

Forty-seven publications comprising 59 field investigations were included. The pooled PPE of schistosomiasis, schistosomiasis mansoni and schistosomiasis haematobium in humans were 27.5% [95% confidence interval (*CI*): 24.0–31.1%], 25.6% (95% *CI*: 19.9–31.3%), and 28.8% (95% *CI*: 23.4–34.3%), respectively. The snails showed an overall infection rate of 8.6% (95% *CI*: 7.7–9.4%), with 12.1% (95% *CI*: 9.9–14.2%) in the *Biomphalaria* spp. snails and 6.9% (95% *CI*: 5.7–8.1%) in the *Bulinus* spp. snails. The correlation coefficient was 0.3 (95% *CI*: 0.01–0.5%, *P* < 0.05) indicating that the two variables, i.e. all intermediate host snails on the one hand and the human host on the other, were positively correlated.

**Conclusions:**

The prevalence rate of *S. mansoni* and *S. haematobium* is still high in endemic areas. Given the significant, positive correlation between the prevalence of schistosomes in humans and the intermediate snail hosts, more attention should be paid to programme integration of snail surveillance in future.

**Graphical Abstract:**

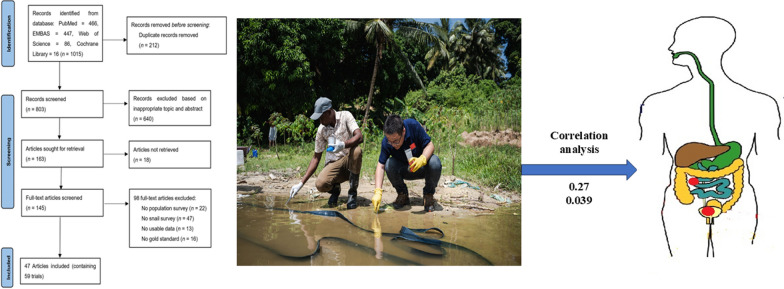

**Supplementary Information:**

The online version contains supplementary material available at 10.1186/s40249-024-01233-0.

## Background

Schistosomiasis (bilharziasis), a parasitic disease caused by different species of trematode worms, is prevalent in 78 countries across Asia, Latin America, the Middle East and Africa [1]. Among the six schistosome species infective for humans, *Schistosoma japonicum*, *S. mansoni* and *S. haematobium* are the major species, with extensive distributions and considerable disease burdens [2]. The previous two cause intestinal schistosomiasis, whereas *S. haematobium* is responsible for the urogenital form of the disease. Over 250 million people worldwide are infected, with more than 95% occurring in sub-Saharan Africa, primarily attributed to *S. mansoni* and *S. haematobium* [3–5]*.*

The life cycle of this parasite involves an intermediate snail host and a definitive mammalian host. Infection of the latter occurs through contact with freshwater contaminated by schistosome cercariae (the infectious form of schistosomes) released from infected snails. After maturing into adult male and female worms, the parasites reproduce and release eggs that are excreted into the aquatic environment with faeces (*S. mansoni, S. japonicum*) or urine (*S. haematobium*). These eggs hatch and infect certain freshwater snails, where the parasites undergo asexually multiplication and finally develop into cercariae [4, 6]. The endemicity of schistosomiasis is related to the presence of appropriate intermediate host snails, with *Biomphalaria* spp. serving as intermediate hosts of *S. mansoni* [7, 8] and *Bulinus* spp. of *S. haematobium* [9]*.* These snail species are hermaphroditic, capable of self- or cross-fertilization and widely distributed in Africa, Latin America and the Middle East as well as countries bordering the Indian and the Mediterranean Seas [10].

In 2001, the World Health Assembly (WHA) proposed a global strategy (resolution 54.19) for controlling schistosomiasis through preventive chemotherapy (PC) programmes predominately to school-age children (SAC) in endemic settings, which rapidly achieved remarkably positive results [3, 11, 12]. This resolution recommends regular treatments by mass drug administration (MDA) with praziquantel (PZQ) which still remains the solely available drug for treating and controlling schistosomiasis [13]. Although PZQ has been the cornerstone for morbidity control of schistosomiasis since the mid-1980s, snail control is not routinely implemented due to higher cost and not considered as important as before PZQ became widely available [14]. However, in the last decade there has been a revival for snail control and it is recommended to also integrate other measures, such as water, sanitation and hygiene (WASH) and health education to accelerate schistosomiasis elimination in Africa [5, 15, 16]. Due to the high heterogeneity of schistosomiasis infection rates in different countries, scientific and reliable data are needed to provide support for improving resource utilization and adjusting countermeasures.

A malacological survey is an important component of epidemiological survey of schistosomiasis. The finding of infected snails and the identification of miracidia in aquatic samples are the two vital components of a malacological survey [17]. However, little attention has been given to malacological approaches as one of the controls of schistosomiasis [18]. Morbidity reduction and ultimate elimination via integrated control actions have been the targets of the existing schistosomiasis control progammes. It has been suggested that precise identification of the infections in humans and intermediate snail hosts, the definitive and intermediate hosts, respectively, is of paramount use in achieving these goals. The vast majority of researches on schistosomiasis have been given major emphasis on disease prevalence and intensity of infection among human populations. Although previous studies reported that snail infections are supposed to indicate the infection rate and magnitude of human schistosomiasis, efforts to identify and target the intermediate snail hosts in endemic areas are apparently overlooked [19]. Therefore, integrating snail distribution with human infection data is quite useful for the ongoing control program.

In 2019, approximately 61.8 million SAC and 1.1 million adults worldwide received PC [20], resulting in significant reduction of schistosomiasis-associated mortality and morbidity [11, 21]. Although the guideline for PC have been revised for the endemic settings, the criteria for frequency of MDA and assessment of effectiveness of interventions are mainly based on the prevalence in SAC and have largely remained the same for the last two decades [22, 23]. In February 2022, World Health Organization (WHO) released updated guideline for the control and elimination [24], and interruption of transmission of human schistosomiasis where feasible in selected endemic countries by 2030 [25]. In the new guideline, the target population for PC has been expanded from SAC to all age groups at risk of infection, thus increasing the demand of PZQ tablets considerably [26]. Considering the gap between available PZQ and requirements, the heterogeneity of schistosomiasis due to the different species and ongoing interventions [27], alternative approaches to assess endemicity of schistosomiasis in a community, especially in Africa, are urgently needed [28].

Previous studies have highlighted the role of the intermediate host for spread the disease [29]. But the relationship of infection rates between intermediate host and humans remains unclear. This systematic review aimed to identify the correlation of prevalence rates in snail hosts and humans for the two major forms of human schistosomiasis, schistosomiasis mansoni and schistosomiasis haematobium, by investigating what has been published on the matter as this could assist policy-making for campaigns against schistosomiasis.

## Methods

### Literature search strategy and selection criteria

Relevant publications from the literature published from January 1991 to December 2022 were searched in public databases (PubMed, Web of Science, Science Direct). The Scopus and Cochrane databases were also included but did not yield additional data. The following keywords and combinations were used in the search: ((*Schistosoma mansoni*) OR (*S. mansoni*) OR (*Schistosoma haematobium*) OR (*S. haematobium*)) AND ((schistosome intermediate host) OR (freshwater snails) OR (malacological survey) OR (*Biomphalaria*) OR (*Bulinus*)) AND (human) AND ((infection rate) OR (prevalence) OR (positive rate)), without language restrictions. The references of the retrieved literatures were scrutinized and screened to capture any study potentially overlooked during the electronic search process (Additional file 1). Titles and abstracts of papers retrieved were manually screened to remove irrelevant references and the full texts of potentially relevant papers were reviewed further [30]. This process was conducted independently by three reviewers (XYW, KY and JX). The systematic review and selection of relevant literature was done according to the Preferred Reporting Items for Systematic Reviews and Meta-analysis (PRISMA) guidelines [31] with PROSPERO (CRD42023471218).

Relevant studies fulfilling the inclusion and exclusion criteria were enrolled. The main inclusion criterion was that retrospective, descriptive or observational studies should focus on the intermediate and the definitive hosts of *S. mansoni* and *S. haematobium* without restrictions. Exclusion criteria included exclusively dealing with intermediate host snails or exclusively with definitive hosts, non-human schistosomes and/or other trematode species. The detailed inclusion and exclusion criteria are described in the Additional file 1.

Data were extracted by QL and YLL and reviewed by SYG. Data were extracted using a standardised form. Discrepancies were resolved by consensus. Where the same study was described in more than one publication, the publication with the highest sample size and most detailed information was used, supplemented by the other publications. A study consists of multiple surveys, with data collected separately for each survey. If a study provided data for one year or more, we used the starting year or year with the most detailed information. The following information was extracted from the selected literature: name of first author, year of study (the years of the studies included in the review were categorized into three groups, namely; the 1991–2000, 2001–2010 and 2011–2022, to assess the trends of infection rates during the periods), study area (e.g., site and country), study type (e.g., cross-sectional or longitudinal), *Schistosoma* species, target population, number of people assessed, number of positive cases, snail species, number of snails examined, number of infected snails, and diagnostic method(s) used. All extracted data were independently recorded with Microsoft Excel 2016 (Microsoft, Redmond, Washington, USA) by those of the authors involved at this stage.

### Quality assessment

The quality of the selected studies was assessed according to the Joanna Briggs Institute (JBI) prevalence critical appraisal Tool [32]. All included studies were scored using the 10 quality control items suggested by the tool. A score of 1 was given for each fulfilled item, with 0 for each unmet item. The overall quality of each included study was classified based on the total number of scores generated, i.e., 0–3 = low, 4–6 = moderate and 7–10 = high (Additional file 1: Table S1) [33].

According to the scoring results, we selected literatures with high scores for inclusion in the study. We assessed methodological quality and risk of bias for all included studies using RevMan 5.4 (The Cochrane Centre Collaboration, Copenhagen, Denmark), and assessed evidence of publication bias by generating funnel plots [34]. We considered the presence of such a bias risk across the selected studies assessing them by funnel plot as introduced by Light and Pillemer [35]. Briefly: a symmetric funnel shape arises from an acceptable dataset makes publication bias unlikely, while an asymmetrical plot indicates the opposite. Publication bias was evaluated by visual inspection of funnel plots method.

### Statistical analysis

The pooled prevalence estimate (PPE) of schistosomes in humans and snails were pooled using random effect model for meta-analysis from the eligible studies [36]. Forest plot, a tool that sums up information on studies in a figure that gives a visual indication (https://s4be.cochrane.org/blog/2016/07/11/tutorial-read-forest-plot/), was used to estimate the overall pooled effect size with 95% confidence interval (*CI*). The percentage of total variation due to interstudy heterogeneity was evaluated using the *I*^*2*^ measure by RevMan 5.4 software, the values of which at 25%, 50% and 75% are considered low, moderate and high heterogeneity, respectively. Subgroup analysis was further performed based on *Schistosoma* species, snail species, years of studies conducted, population and country to explore the source of heterogeneity. The data analysis was conducted using RevMan software.

The normality distribution of prevalence values of schistosome infections between intermediate host and humans was quantified by the Kolmogorov–Smirnov (K-S) test [37]. The correlation coefficient (*r*) was calculated to assess the strength of the linear relationship between two variables. At *r* ≤ 0.4, the strength of correlation was stratified as weak, at 0.4 < *r* < 0.8 moderate and at *r* ≥ 0.8 strong [38, 39]. Regression analysis was used to calculate the regression coefficient and the regression equation. The F test conducts a significant test of the regression equation. If the *P* value of the overall F test is significant, the regression model predicts the response variable better than the mean of the response [40]. Outliers were tested for using residuals and q-q plots when warranted. Analysis of these variable is not always straightforward and standard linear analysis could be problematic. We present the most common approach to dealing with this problem: a logit, a double arcsine and an exponential transformation of the percentages, following which standard linear association analysis can be conducted on the transformed value. The strength of the linear association is expressed by the coefficient of determination (*R*^*2*^), which ranges from 0 (no linear association) to 1 (perfect linear association, whether positive or negative). All analytical functions were analyzed by the statistical software SPSS 20.0 (International Business Machines Corporation, Armonk, New York, USA). *P* < 0.05 was considered statistically significant.

## Results

### Search results

**Fig. 1:**
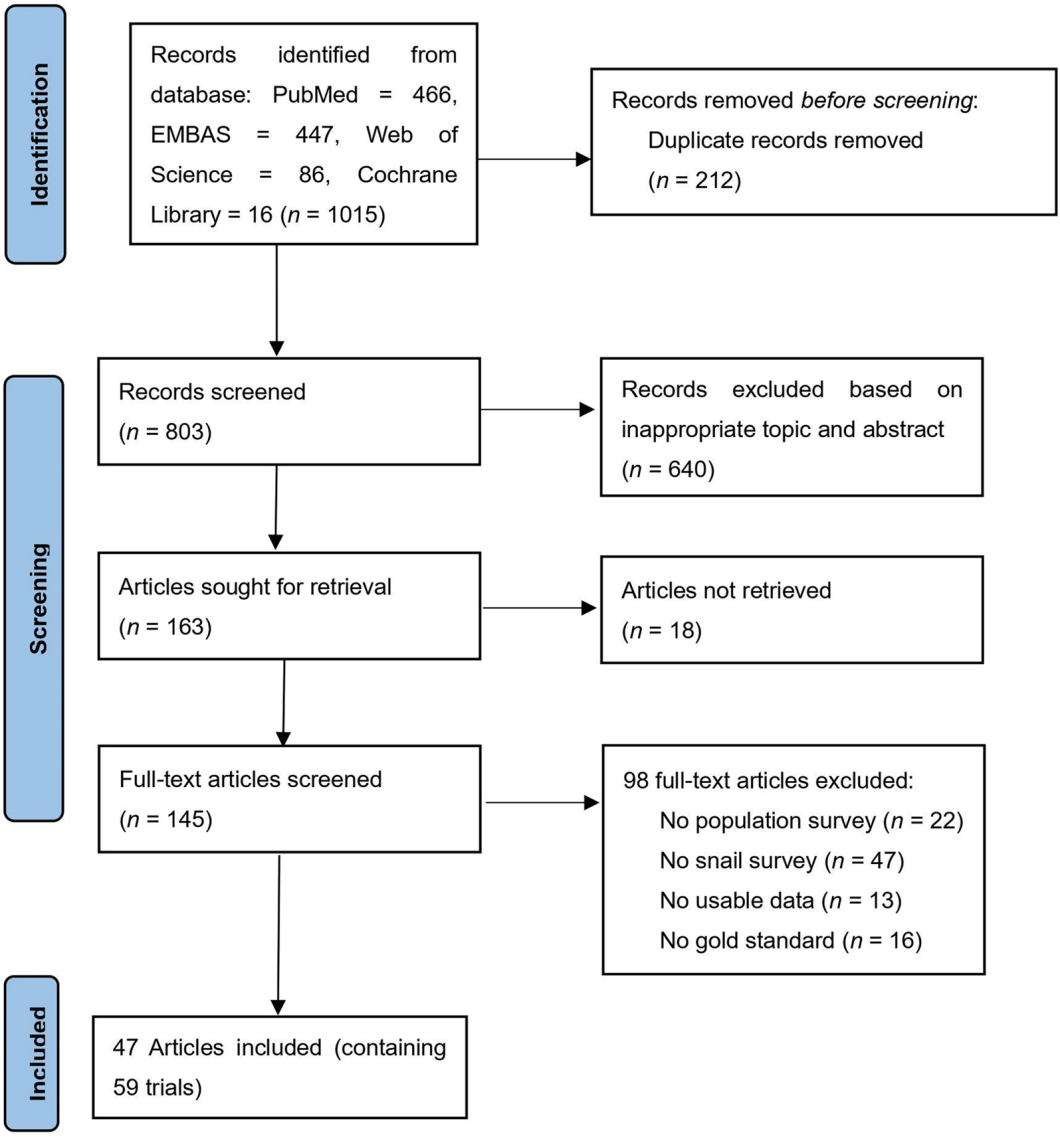
PRISMA flow diagram of studies identified for the study of the correlation of human and snail schistosome infections

An initial number of 1015 relevant studies were identified, 212 of which were removed due to duplications and 640 based on the title and abstract screening. The remaining 145 full-text articles were assessed for eligibility, which led to the exclusion of 98 arriving at a final count of 47 articles containing 59 field investigations of schistosomiasis in human and snail hosts to be used for meta-analysis. The flow diagram of the process is shown in Fig. 1.

The 47 studies [5, 41–87] included in this review had been conducted in 21 countries (Additional file 1: Fig S1), mostly in Africa, with special reference to Ethiopia (*n* = 7), Tanzania (*n* = 5) and Nigeria (*n* = 5), with Brazil (*n* = 4). Among the included 47 studies, 26 focused exclusively on the prevalence of *S. haematobium* in humans and *Bulinus* spp. snails and 13 exclusively on infection rates of *S. mansoni* in humans and *Biomphalaria* spp*.* snails, while the 8 studies included both species in humans and their specific snail hosts. All diagnoses had been carried out by microscopy: in humans for *S. haematobium* after filtration [88] and for *S. mansoni* by Kato-Katz faecal smears [89], while cercarial shedding technique [90] had been used for all snail studies. 35 of the included studies were cross-sectional and 12 longitudinal. According to the JBI prevalence critical appraisal method, all studies covered by this review were deemed to be of high quality with scores ranging from 8 to 9 (Tables 1, 2).

**Table 1:** *Schistosoma haematobium* studies included in the meta-analysis

First author (Published year) [reference no.]	Study year	Population investigated (*n*)	No. infected humans (prevalence, %)	Examined snail species (*n*)	No. infected snails (prevalence, %)	Location	Sampling approach	JBI score
Rudge et al. (2008) [76]	2005	Students (150)	76 (50.7)	*Bulinus globosus* (120)	6 (5.0)	Tanzania	Cross-sectional	8
Léger et al. (2020) [a] [63]	2016–2018	Others (869)	581 (66.9)	*B. globosus, B. truncatus* (2532)	91 (3.6)	Senegal	Longitudinal	9
Léger et al. (2020) [b] [63]	2016–2019	Others (716)	211 (29.5)	*B. umbilicatus* (4694)	6 (0.1)	Senegal	Longitudinal	9
Tchuenté et al. (2018) [79]	2015–2017	Others (1173)	275 (23.4)	*B. camerunensis,* *B. truncates, B. forskalii* (1064)	5 (0.5)	Cameroon	Longitudinal	9
Ivoke et al. (2014) [60]	2012–2013	Students (894)	137 (15.3)	*B. globosus* (308)	62 (20.1)	Nigeria	Longitudinal	9
Vera et al. (1992) [81]	1988	Students (92)	77 (83.7)	*B. senegalensis* (600)	3 (0.5)	Niger	Cross-sectional	8
Medhat et al. (1993) [65]	1993	Others (920)	75 (8.2)	*B. truncates* (1039)	1 (0.1)	Egypt	Cross-sectional	9
Verle et al. (1994) [82]	1994	Community (352)	306 (86.9)	*B. globosus, B. truncatus, B. forskalii, B. senegalensis* (601)	106 (17.6)	Senegal	Cross-sectional	9
Traquinho et al. (1998) [80]	1995	Students (994)	839 (84.4)	*Bulinus* spp. (407)	345 (84.8)	Mozambique	Cross-sectional	9
Pennance et al. (2016) [74]	2014	Students (744)	125 (16.8)	*B. globosus* (1111)	26 (2.3)	Tanzania	Cross-sectional	9
Angelo et al. (2018) [40]	2015–2017	Students (250)	42 (16.8)	*B. nasutus* (4899)	132 (2.7)	Tanzania	Longitudinal	9
Dabo et al. (2015) [50]	2011–2012	Students (1761)	259 (14.7)	*B. globosus, B. truncatus, B. forskalii* (438)	11 (2.5)	Mali	Longitudinal	9
Anyan et al. (2019) [42]	2012–2013	Students (383)	149 (38.9)	*B. truncates* (896)	7 (0.8)	Ghana	Cross-sectional	9
Ofoezie et al. (1997) [a] [72]	1991–1992	Students (128)	51 (39.8)	*B. globosus* (1472)	55 (3.7)	Nigeria	Longitudinal	8
Ofoezie et al. (1997) [b] [72]	1992–1993	Students (99)	33 (33.3)	*B. globosus* (1343)	37 (2.8)	Nigeria	Longitudinal	8
Chimbari et al. (2003) [49]	2001–2002	Others (570)	45 (7.9)	*B. globosus* (120)	4 (3.3)	Zimbabwe	Longitudinal	9
De Clercq et al. (2000) [52]	1997–1999	Community (233)	84 (36.1)	*B. senegalensis, B. truncates, B. forskalii* (257)	15 (5.8)	Senegal	Longitudinal	9
Emejulu et al. (1994) [53]	1990–1992	Community (1773)	736 (41.5)	*B. globosus, B. truncates* (2323)	117 (5.0)	Nigeria	Longitudinal	9
Dahesh et al. (2016) [51]	2016	Students (1285)	52 (4.1)	*B. truncates* (74)	8 (10.8)	Egypt	Cross-sectional	9
Kaiglová et al. (2020) [61]	2018	Community (451)	69 (15.3)	*B. globosus* (68)	6 (8.8)	Kenya	Cross-sectional	8
Okeke et al. (2013) [73]	2012	Students (323)	15 (4.6)	*B. senegalensis, B. globosus* (857)	5 (0.6)	Nigeria	Cross-sectional	9
Chaula et al. (2014) [48]	2013	Students (488)	73 (15.0)	*Bulinis* spp. (46)	6 (13.0)	Tanzania	Cross-sectional	9
Anosike et al. (2006) [41]	2001–2002	Others (2104)	466 (22.2)	*B. globosus, B. truncatus, B. senegalensis* (210)	45 (21.4)	Nigeria	Cross-sectional	9
Mutuku et al. (2011) [70]	2009–2010	Community (777)	336 (43.2)	*B. truncates* (156)	6 (3.9)	Kenya	Cross-sectional	9
Zongo et al. (2012) [83]	2009–2010	Students (648)	138 (21.3)	*B. senegalensis, B. globosus, B. truncates* (291)	13 (4.5)	Burkina Faso	Cross-sectional	9
Krauth et al. (2017) [62]	2014–2015	Community (743)	16 (2.2)	*B. globosus, B. truncates, B. forskalii* (76)	0 (0.0)	Côte d’Ivoire	Cross-sectional	8
Tchuem-Tchuenté et al. (2001) [78]	1999	Community (241)	1 (0.4)	*B. forskalii* (300)	0 (0.0)	Cameroon	Cross-sectional	8
Ibikounlé et al. (2014) [59]	2010–2012	Students (1585)	466 (29.4)	*B. globosus, B. forskalii* (165)	0 (0.0)	Benin	Cross-sectional	9
Poole et al. (2014) [75]	2012	Students (373)	63 (16.9)	*B. globosus* (250)	0 (0.0)	Malawi	Cross-sectional	9
Gbalégba et al. (2017) [54]	2014–2015	Students (2162)	86 (4.0)	*B. senegalensis, B. truncates, B. forskalii* (284)	0 (0.0)	Mauritania	Cross-sectional	9
Assaré et al. (2020) [44]	2016	Students (274)	7 (2.6)	*B. globosus, B. truncates, B. forskalii* (42)	0 (0.0)	Côte d’Ivoire	Cross-sectional	8
Campbell et al. (2017) [47]	2016	Others (338)	96 (28.4)	*B· camerunensis, B· truncates, B· forskalii* (451)	2 (0.4)	Cameroon	Cross-sectional	9
Ndyomugyenyi et al. (2001) [71]	1991–1992	Students (483)	230 (47.6)	*B. globosus, B. nastus, B. africanus* (148)	0 (0.0)	Tanzania	Cross-sectional	9
Arbaji et al. (1998) [43]	1996	Others (5637)	49 (0.9)	*B. truncates* (195)	0 (0.0)	Jordan	Cross-sectional	9
Moser et al. (2022) [69]	2019	Community (258)	101 (39.2)	*B. truncates* (38)	0 (0.0)	Chad	Cross-sectional	9
Mushi et al. (2022) [84]	2021	Students (649)	342 (52.7)	*B. globosus, B. nastus* (947)	18 (1.8)	Tanzania	Cross-sectional	9

JBI = Joanna Briggs Institute; [a] and [b] represent studies carried out in different regions of area country but published in one article

**Table 2:** *Schistosoma mansoni* studies included in the meta-analysis

First author (Published year) [reference no.]	Study year	Population investigated (*n*)	No. infected humans (prevalence, %)	Examined snail Species (*n*)	No. infected snails (prevalence, %)	Location	Sampling approach	JBI score
Ibikounle et al. (2009) [58]	2003–2006	Others (35)	26 (74.3)	*Biomphalaria pfeifferi* (357)	200 (56.0)	Benin	Longitudinal	8
Léger et al. (2020) [c] [63]	2016–2018	Others (671)	80 (11.9)	*Bi. pfeifferi* (407)	9 (2.2)	Senegal	Longitudinal	9
Traquinho et al. (1998)[c] [80]	1995	Students (994)	5 (0.5)	*Biomphalaria* spp. (31)	19 (61.3)	Mozambique	Cross-sectional	9
Dabo et al. (2015)[c] [50]	2011–2012	Students (1491)	22 (1.5)	*Bi. pfeifferi* (189)	2 (1.1)	Mali	Longitudinal	9
Anyan et al. (2019)[c] [42]	2012–2013	Students (383)	222 (58.0)	*Bi. pfeifferi* (780)	14 (1.8)	Ghana	Cross-sectional	9
Chimbari et al. (2003)[c] [49]	2001–2002	Others (464)	32 (6.9)	*Bi. pfeifferi* (42)	2 (4.8)	Zimbabwe	Longitudinal	9
Alebie et al. (2014) [38]	2013	Students (384)	293 (76.3)	*Bi. pfeifferi* (375)	32 (8.5)	Ethiopia	Cross-sectional	9
Amsalu et al. (2015) [39]	2010	Students (384)	172 (44.8)	*Bio. pfeifferi* (31)	1 (3.2)	Ethiopia	Cross-sectional	9
Mengistu et al. (2011) [68]	2007	Community (517)	136 (26.3)	*Biomphalaria* spp. (560)	325 (58.0)	Ethiopia	Cross-sectional	9
Calasans et al. (2018) [46]	2013–2014	Community (232)	7 (3.0)	*Bi. glabrata* (10,270)	912 (8.9)	Brazil	Longitudinal	9
Mekonnen et al. (2012) [66]	2011	Students (403)	106 (26.3)	*Bi. pfeifferi* (80)	2 (2.5)	Ethiopia	Cross-sectional	9
Zongo et al. (2012)[c] [83]	2009–2010	Students (203)	36 (17.7)	*Bi. pfeifferi* (64)	4 (6.3)	Burkina Faso	Cross-sectional	9
Guerra et al. (1991) [57]	1988	Community (162)	91 (56.2)	*Bi. glabrata* (356)	30 (8.4)	Brazil	Cross-sectional	8
Gryseels et al. (1991) [56]	1982	Community (23,955)	6,017 (25.1)	*Bi. pfeifferi* (29,199)	249 (0.9)	Burundi	Longitudinal	9
Massara et al. (2004) [64]	2001–2003	Students (1186)	101 (8.5)	*Bi. glabrata* (2733)	17 (0.6)	Brazil	Cross-sectional	9
Krauth et al. (2017)[c] [62]	2014–2015	Community (743)	7 (1.0)	*Bi. pfeifferi* (43)	0 (0.0)	Côte d’Ivoire	Cross-sectional	9
Assaré et al. (2020)[c] [44]	2016	Students (274)	26 (9.5)	*Bi. pfeifferi* (92)	0 (0.0)	Côte d’Ivoire	Cross-sectional	9
Bekana et al. (2022) [45]	2018–2019	Students (492)	363 (73.8)	*Bi. pfeifferi* (1463)	357 (24.4)	Ethiopia	Cross-sectional	9
Gomes et al. (2022) [d] [55]	2000	Community (2012)	653 (32.5)	*Bi. glabrata* (2214)	357 (16.1)	Brazil	Cross-sectional	9
Gomes et al. (2022) [e] [55]	2010	Community (2459)	409 (16.6)	*Bi. glabrata* (4707)	272 (5.8)	Brazil	Cross-sectional	9
Gomes et al. (2022) [f] [55]	2020	Community (2028)	179 (8.8)	*Bi. glabrata* (1607)	115 (7.2)	Brazil	Cross-sectional	9
Tamir et al. (2022) [77]	2021	Students (421)	20 (4.8)	*Bi. pfeifferi* (27)	2 (7.4)	Ethiopia	Cross-sectional	9
Meleko et al. (2022) [67]	2021	Community (206)	41 (19.9)	*Bi. pfeifferi, Bi. sudanica* (505)	66 (13.1)	Ethiopia	Cross-sectional	9

JBI = Joanna Briggs Institute. [c] represents *S. mansoni* results published in articles that also show *S. haematobium* results; [d], [e] and [f] represent studies carried out in different regions of a country but published in one article

### Subgroup pooled prevalence estimates of human schistosomiasis

**Fig. 2:**
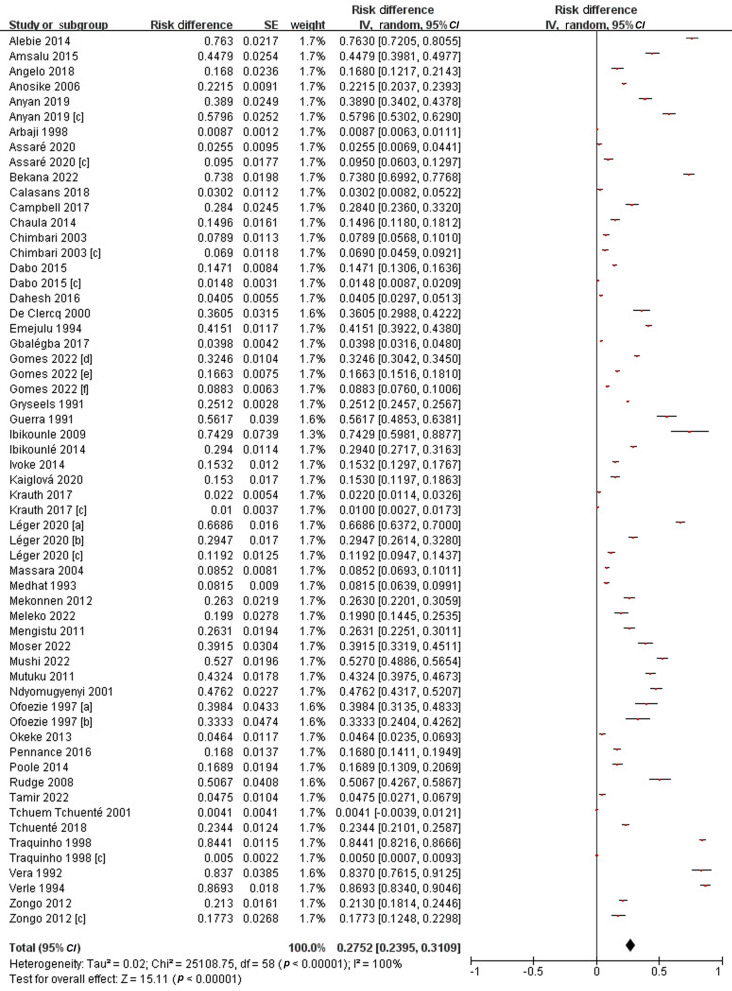
Forest plot diagram showed the prevalence of infecting human schistosomes. * Each red dot represents risk difference of individual studies, and the horizontal line represents the 95% *CI*. The diamond indicates the pooled effect. **a** and **b** represent studies carried out in different regions of a country but published in one article. **c** represents *S. mansoni* results published in articles that also show *S. haematobium* results

In the 47 included articles, a total of 71,019 people were examined and 15,751 of them were found to be infected, either by *S. mansoni* or by *S. haematobium*. The PPE was 27.5% (95% *CI*: 24.0–31.1%), with the prevalence varying from 0.4% to 86.9%, thus showing a high degree of heterogeneity (*I*^*2*^ = 100%, *P* < 0.01) (Fig. 2).

Subgroup analyses were performed based on publication year, areas surveyed, *Schistosoma* species and various populations. Accordingly, the PPE of schistosomiasis in humans was 38.2% (95% *CI*: 30.0–46.4%) from 1991 to 2000; 26.9% (95% *CI*: 20.5–33.4%) from 2001 to 2010; and 22.7% (95% *CI*: 18.1–27.3%) from 2011 to 2022. Geographically, the highest PPE was obtained from Africa 29.0% (95% *CI*: 24.5–33.4%); followed by Latin America 20.3% (95% *CI*: 11.7–28.8%); and only Jordan from West Asia 0.9% (95% *CI*: 0.6–1.1%). Specifically, the PPE of schistosomiasis haematobium in humans was 28.8% (95% *CI*: 23.4–34.3%), while that of schistosomiasis mansoni was 25.6% (95% *CI*: 19.9–31.3%). Among populations, the PPE was 29.3% (95% *CI*: 23.4–35.1%) obtained from students, 26.5% (95% *CI*: 19.0–34.1%) for community populations, and 24.7% (95% *CI*: 14.9–34.5%) for all others (Additional file 1: Fig. S2–S5).

### Subgroup pooled prevalence estimates of infected snails

**Fig. 3:**
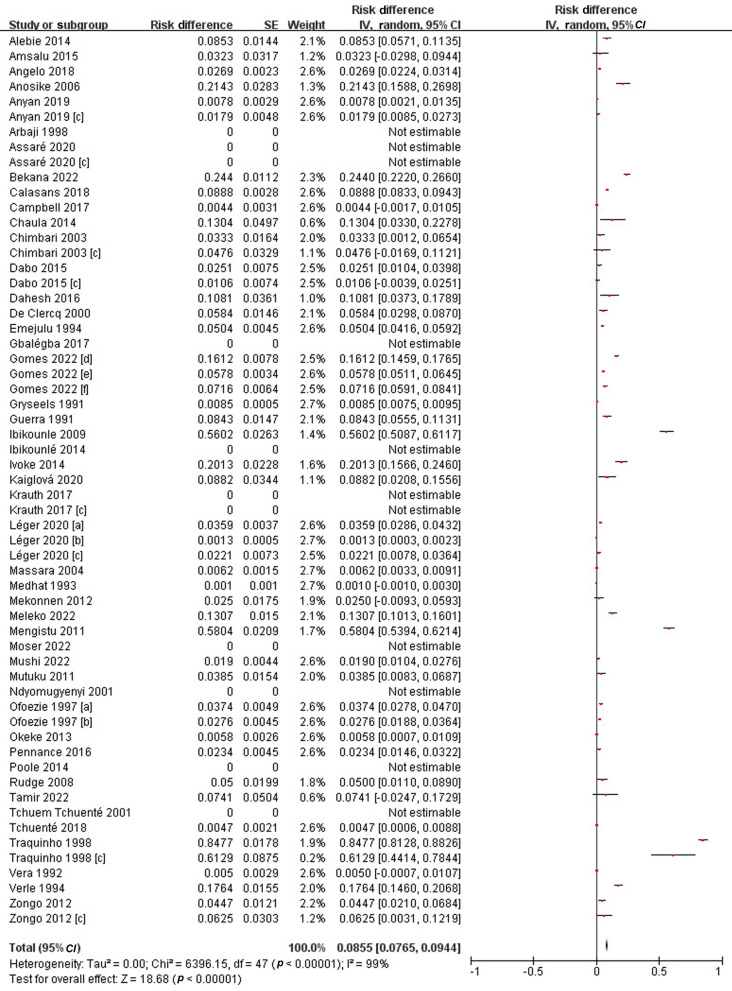
Forest plot diagram showing the prevalence of schistosomes cercariae in snails. * Each red dot represents risk difference of individual studies, and the horizontal line represents the 95% *CI*. The diamond indicates the pooled effect. **a** and **b** represent studies carried out in different regions of a country but published in one article. **c** represents *S. mansoni* results published in articles that also show *S. haematobium* results

A total of 84,954 snails were examined and 4125 of them were infected by either *S. mansoni* or *S. haematobium*. The overall PPE of schistosome cercariae in snails was 8.6% (95% *CI*: 7.7–9.4%). The prevalence extracted from included studies ranged from 0 to 84.8%, with substantial heterogeneity across studies (*I*^*2*^ = 99%, *P* < 0.01) (Fig. 3).

Subgroup analysis was performed using the year of publication, areas surveyed and snail species. From 1991 to 2000, the PPE in snails was 12.9% (95% *CI*: 10.6–15.2%); from 2001 to 2010 it was 14.2% (95% *CI*: 9.2–19.3%); and from 2011 to 2022, it decreased to 5.2% (95% *CI*: 3.8–6.5%). The highest PPE in snails was observed in Africa, i.e. 8.5% (95% *CI*: 7.6–9.4%); followed by Latin America (Brazil) with 7.8% (95% *CI*: 3.5–12.1%); and Asia without infection snails [43]. The infection rate of schistosomes in *Bulinus* spp. was 6.9% (95% *CI*: 5.7–8.1%), while it was 12.1% (95% *CI*: 9.9–14.2%) in *Biomphalaria* spp*.* (Additional file 1: Fig. S6–S8).

### Publication bias

The funnel plot dissymmetry demonstrated the presence of publication bias among intermediate host and definitive host studies included in this meta-analysis (Additional file 1: Fig. S9–S10). A high level of heterogeneity was observed (*I*^*2*^ > 90%), something which could not be reduced through subgroup analysis.

### Correlation analysis between the infection rates in the intermediate host and definitive host

The results of the K-S test indicated that the data didn’t follow a normal distribution, so the rank correlation from a non-parametric correlation analysis was used to describe the degree and direction of the correlation between the two variables. The non-parametric correlation analysis indicated that the correlation was statistically significant. The correlation coefficient r was 0.3 (95% *CI*: − 0.01–0.5, *P* < 0.05) indicating that the two variables of all intermediate host snails and definitive hosts were positively correlated. The non-parametric correlation analysis of different schistosomiasis subgroup indicated that the correlation of *S. haematobium* and *Bulinus* spp*.* was statistically significant (*r* = 0.3, 95% *CI*: − 0.04–0.6, *P* < 0.05), while the correlation of *S. mansoni* and *Biomphalaria* spp*.* was not statistically significant (*r* = 0.3, 95% *CI*: − 0.02–0.7, *P* = 0.17).

The regression test indicated that there was a relationship between the infection rates in humans and snail intermediate hosts. The optimal model equation is obtained by fitting the linear and nonlinear models with two sets of parameters. The equation is Y = 0.001x^3^ − 0.056x^2^ + 1.790x + 20.761 (where y is the infection rate of schistosomes in definitive hosts, and x is the infection rate in intermediate hosts). Through this equation, the known infection rate in snail intermediate hosts can predict the human schistosomiasis infection rate. The results of the *F* test gave *F* = 2.9 (*P* < 0.05), which means that the nonlinear relationship between the explained variable and its predictors was significant. The *R*^*2*^ = 0.14, suggests that the intermediate host snail is a factor affecting the changes in local epidemic of schistosomiasis.

## Discussion

Although schistosomiasis is a vector-borne disease and the appropriate intermediate host snail is regarded as an important factor impacting the distribution and endemicity of schistosomiasis, the correlations of the prevalence of schistosomiasis in definitive host and intermediate host is not well known. To the best of our knowledge, this study is the first systematic review and meta-analysis aiming at exploring the relationship of infection rates of schistosomes between intermediate host and humans worldwide, particularly focusing on *S. haematobium* and *S. mansoni*.

The results of this review indicate a persistently high global infection rate, primarily concentrated in sub-Saharan African countries. The PPE of students was highest in all populations. However, it is worth noting that the prevalence of schistosomiasis in the community and others was almost the same as SAC, indicating that the frequency of MDA against schistosomiasis and assessment of effectiveness of interventions only based on the prevalence in SAC are unreasonable [91, 92]. It supports the recommendation of the WHO’s new guideline for control and elimination of human schistosomiasis to extend PC from SAC to all age groups at risk of schistosome infection, with aims to eliminate schistosomiasis as a public health problem or interrupt the transmission of schistosomiasis in endemic communities. The infection rate of *S. haematobium* and *S. mansoni* in humans, seen in the subgroup analysis, was 28.8% and 25.6% respectively, basically identical with the results obtained by Feleke et al. [93] and Cando et al. [94]. The pooled prevalence of schistosomiasis in humans was 38.2% from 1991 to 2000, 26.9% from 2001 to 2010 and 22.7% from 2011 to 2022, showing a slowly decreasing trend over time. In addition, the demographic differences, the years of investigations conducted, as well as the number of snails in each area, may contribute to the difference detected in prevalence of schistosomiasis in humans across countries.

The overall pooled prevalence of schistosome cercariae was 8.6%, emphasizing the importance of snail control. This finding is very similar to the reports on freshwater snails in Brazil and other meta-analyses [95]. The downward PPE trend in snails from 12.9% in 1991−2000 over 14.2% in 2001−2010 to 5.2% in 2011−2022 confirms the decreasing trend presented by Nwoko et al., who reported that the pooled prevalence of schistosome cercariae decreased from 6.0% in the 1990s to 1.0% in the 2000s [49]. PC together with improved sanitation, environmental modification, better health education and behaviour changes might contribute to the recent lowering infection rates among freshwater snails. The highest pooled prevalence of schistosome cercariae obtained from Africa, with 8.5%, followed by Brazil (7.8%), is in line with the geographical prevalence tendency of schistosomiasis in humans.

The regression test indicated that there was a relationship between human schistosomiasis and the infection rate in the intermediate host. The optimal model equation suggests that the intermediate host snail can be a factor affecting the local variations in human schistosomiasis prevalence. We also found a statistical correlation between the prevalence of all schistosomiasis in intermediate host snails and definitive hosts locally, but the correlation was considered weak as the *r* value was less than 0.4. By subgroup analysis, this correlation existed between the prevalence of *S. haematobium* in humans and infection in *Bulinus* spp. snails, but was not detected between the prevalence of *S. mansoni* in humans and *Biomphalaria* spp*.* snails. This could be explained by many factors influencing human schistosomiasis levels, such as frequency of water contact, human behaviour with respect to water contact, the distance between villages and water bodies, intervention strength, capacity of snail survey and case finding [23, 96]. However, mapping the geographical distribution of schistosomiasis in humans as well as in the snail hosts would benefit targeted interventions in critical areas and support resource allocation.

Snail control, mainly by molluscicides, is the cornerstone of schistosomiasis control before the strategy for morbidity control, and has contributed to many successful control outcomes [5]. Early large-scale global schistosomiasis control programmes also emphasized on snail control. However, snail control had been challenged as excessive mollusciciding was considered to lead environmental pollution, destruct aquatic resources and require high cost [97]. WHO recommends WASH interventions, environmental interventions (water engineering and focal snail control with molluscicides) and behavioural change interventions as essential measures to help reduce transmission of *Schistosoma* spp. in endemic areas [23]. This study is the first meta-analysis to prove that the prevalence of schistosomiasis in humans and snails presented statistically significant relationships, supporting that policymakers should pay more attention to the integration of snail control to the ongoing deworming programmes against schistosomiasis.

There are a few limitations in this study, although valuable information was generated specially on the prevalence of *S. mansoni* and *S. haematobium* among freshwater snails and humans. First, available prevalence data were only obtained from 47 studies in 21countries, accounting for only a part of all endemic settings or countries. The pooled prevalence may not fully represent the real infection status of *S. mansoni* or *S. haematobium* in humans and snails*,* leading to a correlation bias. Second, the study was limited by the conventional microscopic techniques as the shortcomings of less sensitivity of these techniques might have a certain impact on the results. We didn’t include data based on molecular techniques for meta-analysis because most research based on molecular techniques were laboratory-based and the methods have not been completely unified [98]. Third, there are limited data on the prevalence of schistosomiasis in West Asia. Some surveys done in Asia could not be included in this analysis because the data were not readily available for both humans and snails. More high-quality research, e.g., high-sensitivity diagnostics is needed to assess whether data obtained from snail survey can be used to guide interventions against schistosomiasis.

## Conclusions

Our findings showed that the overall PPE of either *S. haematobium* or *S. mansoni* in human host was 27.5% and the prevalence of schistosome cercariae was 8.6%, highlighting the need of sustained PC programme and snail control. The prevalence of schistosomiasis in humans and snails presented statistically significant relationships, so the distributions and strengths of infection in the intermediate host snail can be used as an indicator of the level of schistosomiasis risk. Further studies are needed to understand the ecology and transmission of the parasite between the snails and definitive hosts. In addition, policymakers should pay more attention to integration of snail control strategies to the ongoing de-worming programmes against schistosomiasis. This analysis has laid the foundation for the follow-up work and providing a scientific basis for decision-making.

### Supplementary Information

Additional file 1. Table S1. The Joanna Briggs Institute (JBI) Prevalence Critical Appraisal Tool. Figure S1. Global distribution showing country location of included studies. Figure S2. Forest plot of subgroup PPE analysis of infection in the students, community and others. Figure S3. Forest plot of subgroup PPE analysis of infection in different years. Figure S4. Forest plot of subgroup PPE analysis of infection in Africa, South America and Asia. Figure S5. Forest plot of subgroup PPE analysis of infection in the *S. mansoni* and *S. haematobium*. Figure S6. Forest plot of subgroup PPE analysis of snail infectivity indifferent years. Figure S7. Forest plot of subgroup PPE analysis of infectivity in the Africa, South America and Asia. Figure S8. Forest plot of subgroup PPE analysis of infection in the *Biomphalaria* spp. and *Bulinus* spp. Figure S9. Funnel plot with 95% confidence limit showing publication bias across studies on the prevalence of *S. mansoni* and *S. haematobium*. Figure S10. Funnel plot with 95% confidence limit showing publication bias across studies on the prevalence of *S. mansoni* and *S. haematobium* among freshwater snails.

## Data Availability

All datasets generated and analysed, including the search strategy, list of the included and excluded studies, data extracted, and quality assessment, are available in the Article and on request from the corresponding author Jing Xu.
