# Supplementary material for: Prevalence and correlations of schistosomiasis mansoni and schistosomiasis haematobium among humans and intermediate snail hosts: a systematic review and meta-analysis
Source: Infect Dis Poverty. 2024 Sep 2;13:63. doi: 10.1186/s40249-024-01233-0 (PMC11367875; doi:10.1186/s40249-024-01233-0)
Supplement: Supplementary file 1 — Additional file 1. Table S1. The Joanna Briggs Institute (JBI) Prevalence Critical Appraisal Tool. Figure S1. Global distribution showing country location of included studies. Figure S2. Forest plot of subgroup PPE analysis of infection in the students, community and others. Figure S3. Forest plot of subgroup PPE analysis of infection in different years. Figure S4. Forest plot of subgroup PPE analysis of infection in Africa, South America and Asia. Figure S5. Forest plot of subgroup PPE analysis of infection in the S. mansoni and S. haematobium. Figure S6. Forest plot of subgroup PPE analysis of snail infectivity indifferent years. Figure S7. Forest plot of subgroup PPE analysis of infectivity in the Africa, South America and Asia. Figure S8. Forest plot of subgroup PPE analysis of infection in the Biomphalaria spp. and Bulinus spp. Figure S9. Funnel plot with 95% confidence limit showing publication bias across studies on the prevalence of S. mansoni and S. haematobium. Figure S10. Funnel plot with 95% confidence limit showing publication bias across studies on the prevalence of S. mansoni and S. haematobium among freshwater snails. [file 40249_2024_1233_MOESM1_ESM.docx]

Table of Contents

Literature search strategy 1

[Selection of Primary Study 2](#_Toc160985389)

[1. Inclusion criteria 2](#_Toc160985390)

[2. Exclusion criteria 2](#_Toc160985391)

[Quality Assessment Report of Articles Included in the Review 3](#_Toc160985392)

[Global map showing country location of included studies 7](#_Toc160985392)

[Forest plot of subgroup PPE analysis of infecting human schistosomes 8](#_Toc160985393)

[Forest plot of subgroup PPE analysis of infection in the students, community and others 8](#_Toc160985394)

[Forest plot of subgroup PPE analysis of infection in different years 9](#_Toc160985395)

[Forest plot of subgroup PPE analysis of infection in Africa, South America and Asia 10](#_Toc160985396)

[Forest plot of subgroup PPE analysis of infection in the *S. mansoni* and *S. haematobium* 11](#_Toc160985397)

[Forest plot of subgroup PPE analysis of schistosomes cercariae in snails 12](#_Toc160985398)

[Forest plot of subgroup PPE analysis of snail infectivity indifferent years 12](#_Toc160985399)

[Forest plot of subgroup PPE analysis of infectivity in the Africa, South America and Asia 13](#_Toc160985399)

[Forest plot of subgroup PPE analysis of infection in the *Biomphalaria spp.* and *Bulinus* spp. 14](#_Toc160985400)

[Publication bias among intermediate host and definitive host studies 15](#_Toc160985401)

[Funnel plot with 95% confidence limit showing publication bias across studies on the prevalence of *S. mansoni* and *S. haematobium* 15](#_Toc160985402)

[Funnel plot with 95% confidence limit showing publication bias across studies on the prevalence of *S. mansoni* and *S. haematobium* among freshwater snails 16](#_Toc160985403)

# 1. Literature search strategy

PubMed: ((Schistosoma mansoni) OR (S. mansoni) OR (Schistosoma haematobium) OR (S. haematobium)) AND ((schistosome intermediate host) OR (freshwater snails) OR (malacological survey) OR (Biomphalaria) OR (Bulinus)) AND (human) AND ((infection rate) OR (prevalence) OR (positive rate)) AND (("1991/01"[Date - Publication] : "2022/12"[Date - Publication]))

Science Direct: (((Schistosoma haematobium and ('infection rate' or prevalence)) and (Bulinus and positive rate))) and (((Schistosoma mansoni) and ('infection rate' or prevalence)) and (Biomphalaria and positive rate)))

Web of Science: ((Schistosoma haematobium and (infection rate) and (Bulinus and positive rate)) and ((Schistosoma mansoni) and (infection rate) and (Biomphalaria and positive rate))

The Scopus and Cochrane databases were also included but did not yield additional data.

# 2. Selection of Primary Study

## 2.1 Inclusion criteria

- Retrospective, descriptive or observational studies;
- Papers with focus on the intermediate and the definitive hosts of *S. mansoni* and *S. haematobium* without restrictions;
- Diagnosis in humans and snails done by parasitological methods to ensure comparability between studies;
- Original survey data of indicators of infection rates in humans, livestock and snails.
- Studies that reported infection in snails that had been sampled from the field and not laboratory infected snails.
- Parasitological and malacological surveys are carried out simultaneously in one area.
- Since the presence of non-human schistosome cercaria may be detected by etiological detection of snail, inclusion studies must identify schistosomiasis patients.

## 2.2 Exclusion criteria

- Studies exclusively dealing with intermediate host snails or exclusively with definitive hosts;
- Studies on non-human schistosomes and/or other trematode species;
- Repeated reports (only the first published reference was selected);
- Studies without prevalence data;
- Review or meta-analysis articles.

# 3. Quality Assessment Report of Articles Included in the Review

The quality of the included articles was assessed using the Joanna Briggs Institute (JBI) Prevalence Critical Appraisal Tool. The tool assessed each article on the following:

1. Sample representative of the target population
2. Study participants recruited in an appropriate way
3. Sample size adequate
4. Study subjects and setting described in detail
5. Data analysis was conducted with enough coverage of the identified sample
6. Validity of methods used to identify the condition of interest
7. Condition measured in a standard, reliable way for all participants
8. Appropriate statistical analysis
9. Adequacy of response rate; were low response rates managed?
10. Sub-population of interest identified using objective criteria

Table S1. The Joanna Briggs Institute (JBI) Prevalence Critical Appraisal Tool

| **First Author and Year** | **Was the sample representative of the target population?** | **Were study participants recruited in an appropriate way?** | **Was the sample size adequate?** | **Were the study subjects and setting described in detail?** | **Was data analysis conducted with sufficient coverage of the identified sample?** | **Were valid methods used for the identification of the condition?** | **Was the condition measured in a standard, reliable way for all participants?** | **Was there appropriate statistical analysis?** | **Was the response rate adequate, and if not, was the low response rate managed appropriately?** | **Were target subpopulations identified using objective criteria?** | **Quality score** |
| --- | --- | --- | --- | --- | --- | --- | --- | --- | --- | --- | --- |
| Rudge et al., 2008 | Yes | Yes | No | Yes | Yes | Yes | Yes | Yes | NA | Yes | 8 |
| Léger et al., 2020 [a] | Yes | Yes | Yes | Yes | Yes | Yes | Yes | Yes | NA | Yes | 9 |
| Léger et al., 2020 [b] | Yes | Yes | Yes | Yes | Yes | Yes | Yes | Yes | NA | Yes | 9 |
| Tchuenté et al., 2018 | Yes | Yes | Yes | Yes | Yes | Yes | Yes | Yes | NA | Yes | 9 |
| Ivoke et al., 2014 | Yes | Yes | Yes | Yes | Yes | Yes | Yes | Yes | NA | Yes | 9 |
| Vera et al., 1992 | Yes | Yes | No | Yes | Yes | Yes | Yes | Yes | NA | Yes | 8 |
| Medhat et al., 1993 | Yes | Yes | Yes | Yes | Yes | Yes | Yes | Yes | NA | Yes | 9 |
| Verle et al., 1994 | Yes | Yes | Yes | Yes | Yes | Yes | Yes | Yes | NA | Yes | 9 |
| Traquinho et al., 1998 | Yes | Yes | Yes | Yes | Yes | Yes | Yes | Yes | NA | Yes | 9 |
| Pennance et al., 2016 | Yes | Yes | Yes | Yes | Yes | Yes | Yes | Yes | NA | Yes | 9 |
| Angelo et al., 2018 | Yes | Yes | Yes | Yes | Yes | Yes | Yes | Yes | NA | Yes | 9 |
| Dabo et al., 2015 | Yes | Yes | Yes | Yes | Yes | Yes | Yes | Yes | NA | Yes | 9 |
| Anyan et al., 2019 | Yes | Yes | Yes | Yes | Yes | Yes | Yes | Yes | NA | Yes | 9 |
| Ofoezie et al., 1997 [a] | Yes | Yes | No | Yes | Yes | Yes | Yes | Yes | NA | Yes | 8 |
| Ofoezie et al., 1997 [b] | Yes | Yes | No | Yes | Yes | Yes | Yes | Yes | NA | Yes | 8 |
| Chimbari et al., 2003 | Yes | Yes | Yes | Yes | Yes | Yes | Yes | Yes | NA | Yes | 9 |
| De Clercq et al., 2000 | Yes | Yes | Yes | Yes | Yes | Yes | Yes | Yes | NA | Yes | 9 |
| Emejulu et al., 1994 | Yes | Yes | Yes | Yes | Yes | Yes | Yes | Yes | NA | Yes | 9 |
| Dahesh et al., 2016 | Yes | Yes | Yes | Yes | Yes | Yes | Yes | Yes | NA | Yes | 9 |
| Kaiglová et al., 2020 | Yes | Yes | No | Yes | Yes | Yes | Yes | Yes | NA | Yes | 8 |
| Okeke et al., 2013 | Yes | Yes | Yes | Yes | Yes | Yes | Yes | Yes | NA | Yes | 9 |
| Chaula et al., 2014 | Yes | Yes | Yes | Yes | Yes | Yes | Yes | Yes | NA | Yes | 9 |
| Anosike et al., 2006 | Yes | Yes | Yes | Yes | Yes | Yes | Yes | Yes | NA | Yes | 9 |
| Mutuku et al., 2011 | Yes | Yes | Yes | Yes | Yes | Yes | Yes | Yes | NA | Yes | 9 |
| Zongo et al., 2012 | Yes | Yes | Yes | Yes | Yes | Yes | Yes | Yes | NA | Yes | 9 |
| Krauth et al., 2017 | Yes | Yes | No | Yes | Yes | Yes | Yes | Yes | NA | Yes | 8 |
| Tchuem Tchuenté et al., 2001 | Yes | Yes | No | Yes | Yes | Yes | Yes | Yes | NA | Yes | 8 |
| Ibikounlé et al., 2014 | Yes | Yes | Yes | Yes | Yes | Yes | Yes | Yes | NA | Yes | 9 |
| Poole et al., 2014 | Yes | Yes | Yes | Yes | Yes | Yes | Yes | Yes | NA | Yes | 9 |
| Gbalégba et al., 2017 | Yes | Yes | Yes | Yes | Yes | Yes | Yes | Yes | NA | Yes | 9 |
| Assaré et al., 2020 | Yes | Yes | No | Yes | Yes | Yes | Yes | Yes | NA | Yes | 8 |
| Campbell et al. 2017 | Yes | Yes | Yes | Yes | Yes | Yes | Yes | Yes | NA | Yes | 9 |
| Ndyomugyenyi et al., 2001 | Yes | Yes | Yes | Yes | Yes | Yes | Yes | Yes | NA | Yes | 9 |
| Arbaji et al., 1998 | Yes | Yes | Yes | Yes | Yes | Yes | Yes | Yes | NA | Yes | 9 |
| Moser et al., 2022 | Yes | Yes | Yes | Yes | Yes | Yes | Yes | Yes | NA | Yes | 9 |
| Mushi et al., 2022 | Yes | Yes | Yes | Yes | Yes | Yes | Yes | Yes | NA | Yes | 9 |
| Ibikounle et al., 2009 | Yes | Yes | No | Yes | Yes | Yes | Yes | Yes | NA | Yes | 8 |
| Léger et al., 2020 | Yes | Yes | Yes | Yes | Yes | Yes | Yes | Yes | NA | Yes | 9 |
| Traquinho et al., 1998 | Yes | Yes | Yes | Yes | Yes | Yes | Yes | Yes | NA | Yes | 9 |
| Dabo et al., 2015 | Yes | Yes | Yes | Yes | Yes | Yes | Yes | Yes | NA | Yes | 9 |
| Anyan et al., 2019 | Yes | Yes | Yes | Yes | Yes | Yes | Yes | Yes | NA | Yes | 9 |
| Chimbari et al., 2003 | Yes | Yes | Yes | Yes | Yes | Yes | Yes | Yes | NA | Yes | 9 |
| Alebie et al., 2014 | Yes | Yes | Yes | Yes | Yes | Yes | Yes | Yes | NA | Yes | 9 |
| Amsalu et al., 2015 | Yes | Yes | Yes | Yes | Yes | Yes | Yes | Yes | NA | Yes | 9 |
| Mengistu et al., 2011 | Yes | Yes | Yes | Yes | Yes | Yes | Yes | Yes | NA | Yes | 9 |
| Calasans et al., 2018 | Yes | Yes | Yes | Yes | Yes | Yes | Yes | Yes | NA | Yes | 9 |
| Mekonnen et al., 2012 | Yes | Yes | Yes | Yes | Yes | Yes | Yes | Yes | NA | Yes | 9 |
| Zongo et al., 2012 | Yes | Yes | Yes | Yes | Yes | Yes | Yes | Yes | NA | Yes | 9 |
| Guerra et al., 1991 | Yes | Yes | No | Yes | Yes | Yes | Yes | Yes | NA | Yes | 8 |
| Gryseels et al., 1991 | Yes | Yes | Yes | Yes | Yes | Yes | Yes | Yes | NA | Yes | 9 |
| Massara et al., 2004 | Yes | Yes | Yes | Yes | Yes | Yes | Yes | Yes | NA | Yes | 9 |
| Krauth et al., 2017 | Yes | Yes | Yes | Yes | Yes | Yes | Yes | Yes | NA | Yes | 9 |
| Assaré et al., 2020 | Yes | Yes | Yes | Yes | Yes | Yes | Yes | Yes | NA | Yes | 9 |
| Bekana et al., 2022 | Yes | Yes | Yes | Yes | Yes | Yes | Yes | Yes | NA | Yes | 9 |
| Gomes et al., 2022 [d] | Yes | Yes | Yes | Yes | Yes | Yes | Yes | Yes | NA | Yes | 9 |
| Gomes et al., 2022 [e] | Yes | Yes | Yes | Yes | Yes | Yes | Yes | Yes | NA | Yes | 9 |
| Gomes et al., 2022 [f] | Yes | Yes | Yes | Yes | Yes | Yes | Yes | Yes | NA | Yes | 9 |
| Tamir et al., 2022 | Yes | Yes | Yes | Yes | Yes | Yes | Yes | Yes | NA | Yes | 9 |
| Meleko et al., 2022 | Yes | Yes | Yes | Yes | Yes | Yes | Yes | Yes | NA | Yes | 9 |

**Reference**

1. Munn Z, Moola S, Riitano D, Lisy K. The development of a critical appraisal tool for use in systematic reviews addressing questions of prevalence. Int J Health Policy Manag. 2014;3(3):123-8.


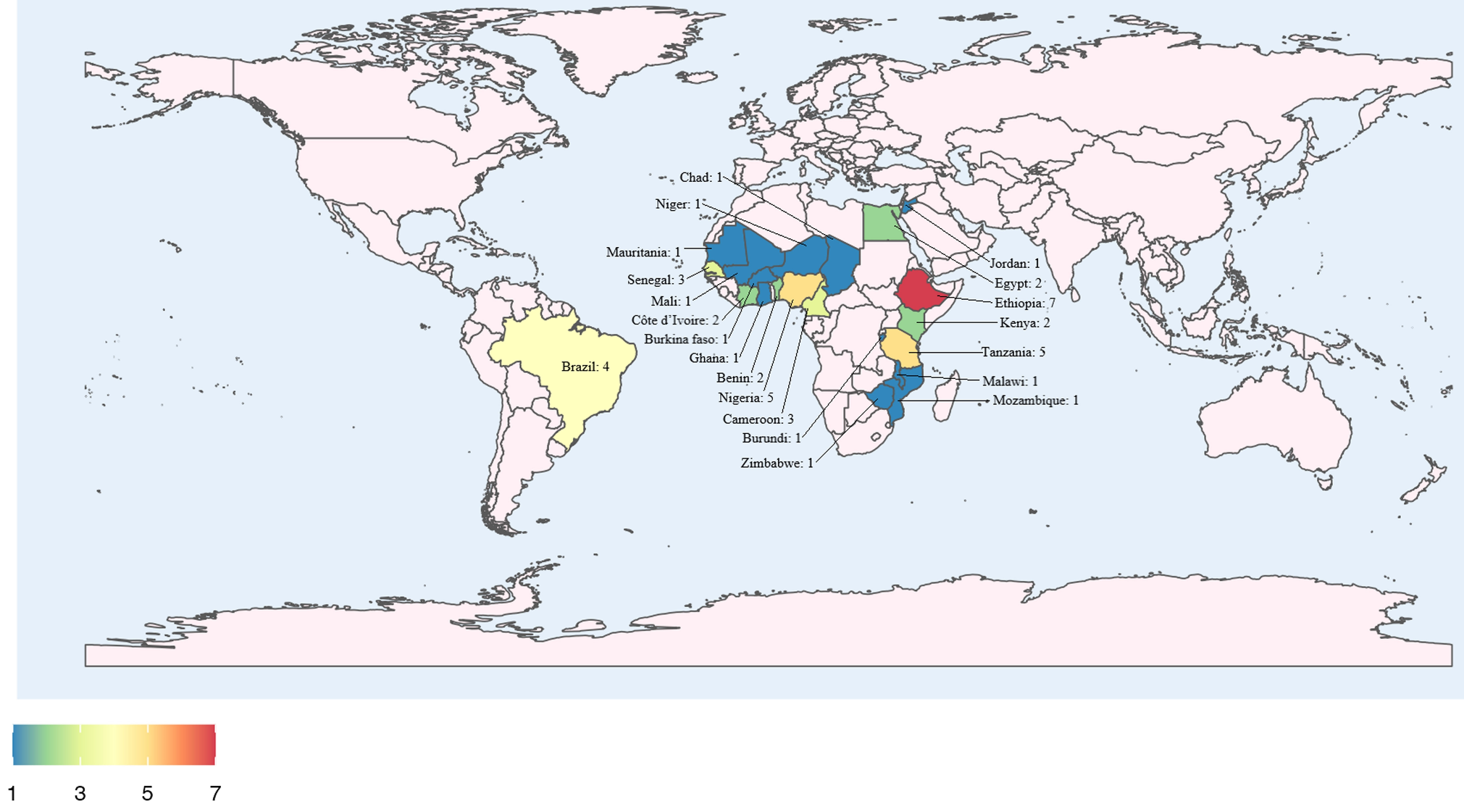


**Figure S1. Global distribution showing country location of included studies**

*The global map comes from inside the R software

# Forest plot of subgroup PPE analysis of infecting human schistosomes


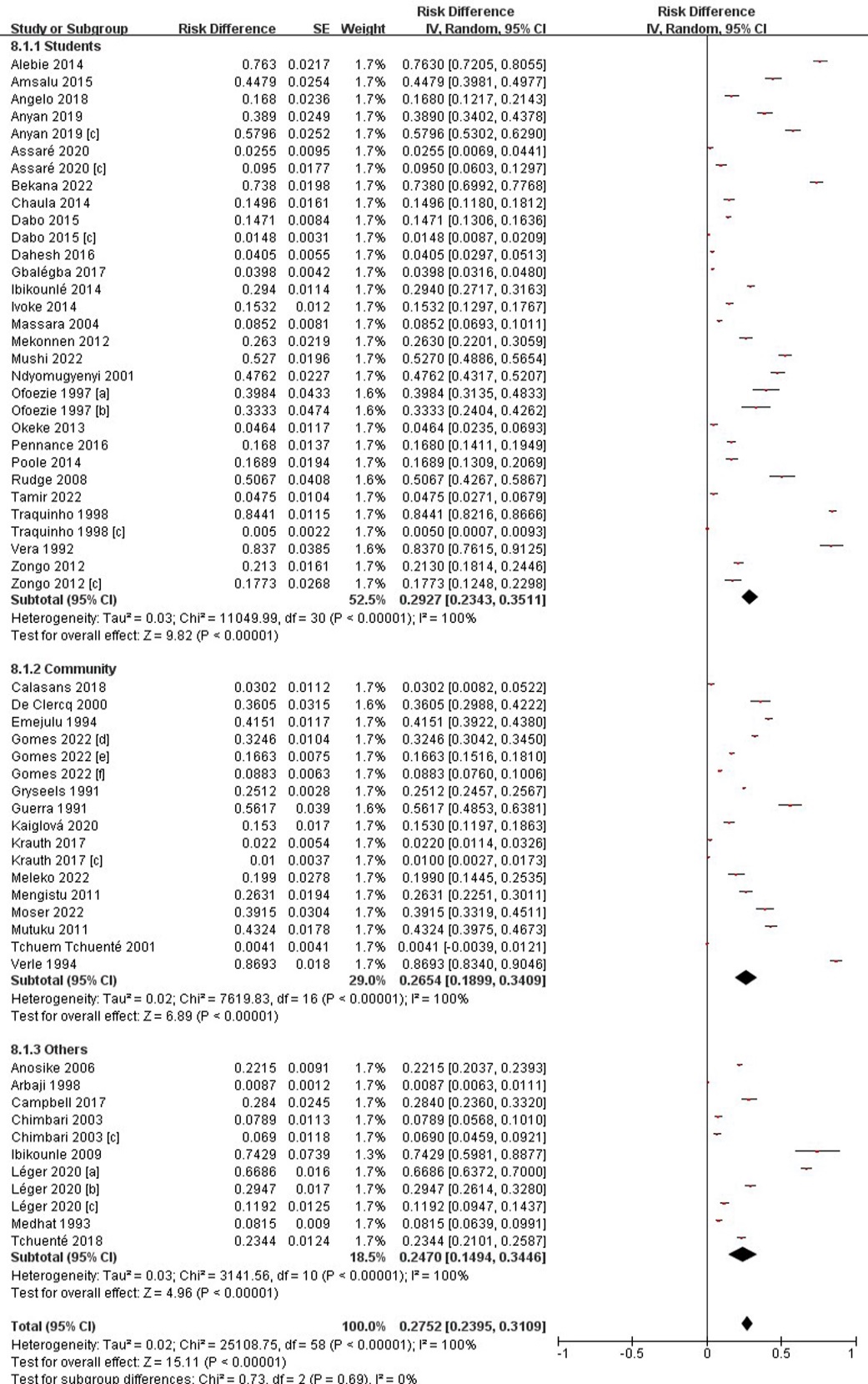


## Figure S2. Forest plot of subgroup PPE analysis of infection in the students, community and others


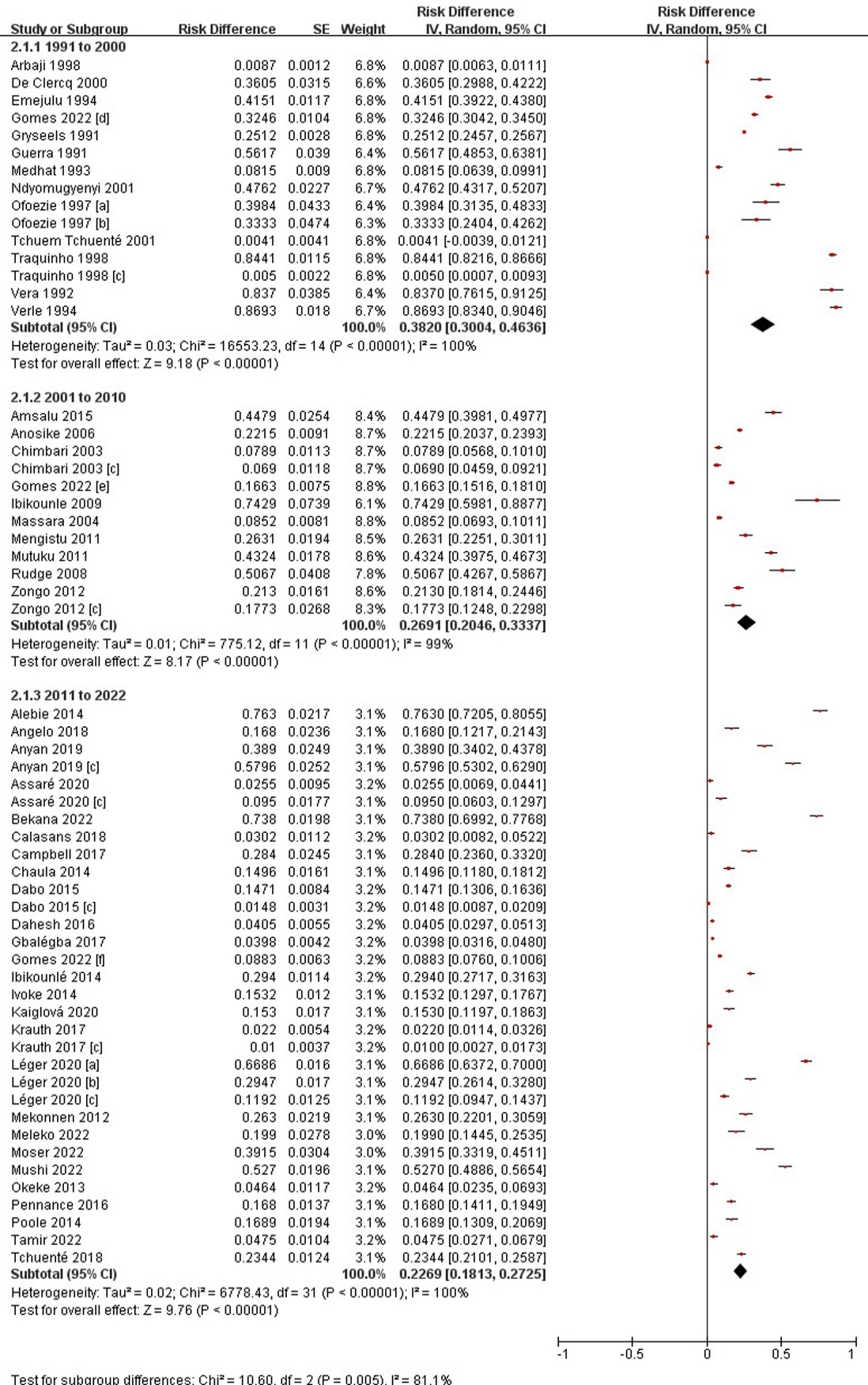


## Figure S3. Forest plot of subgroup PPE analysis of infection in different years


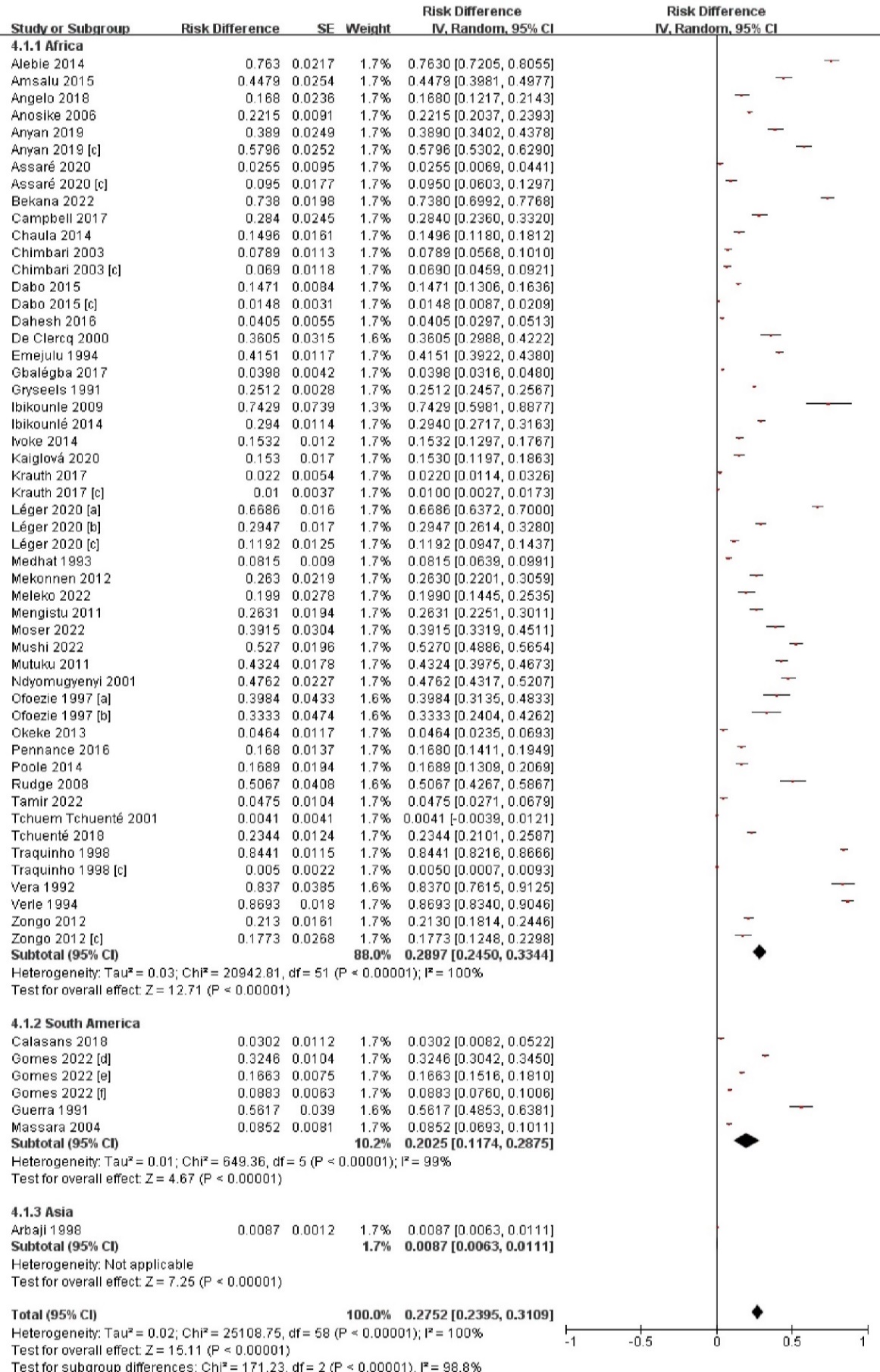


## Figure S4. Forest plot of subgroup PPE analysis of infection in Africa, South America and Asia


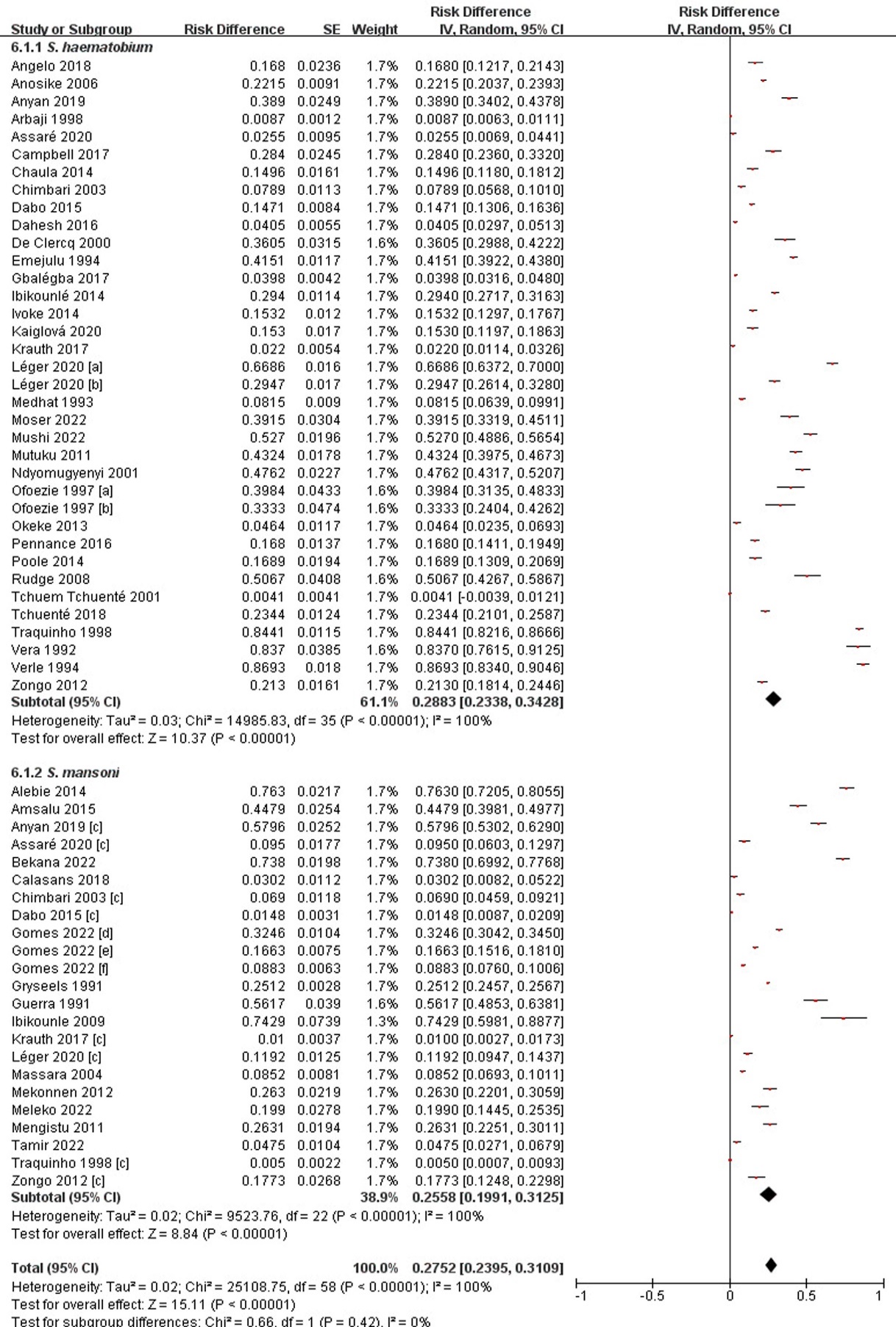


## Figure S5. Forest plot of subgroup PPE analysis of infection in the *S. mansoni* and *S. haematobium*

# Forest plot of subgroup PPE analysis of schistosomes cercariae in snails


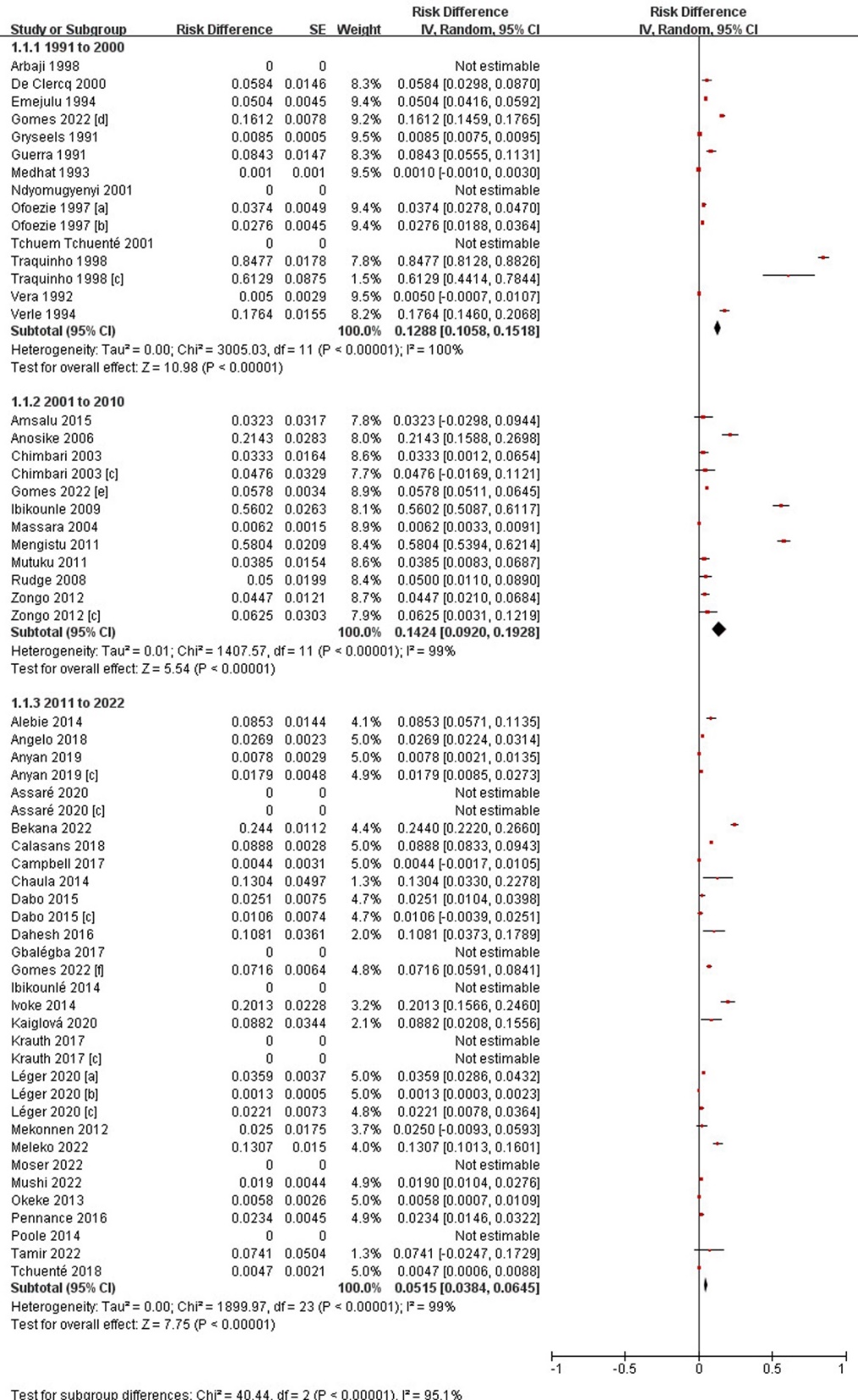


**Figure S6. Forest plot of subgroup PPE analysis of snail infectivity indifferent years**

##


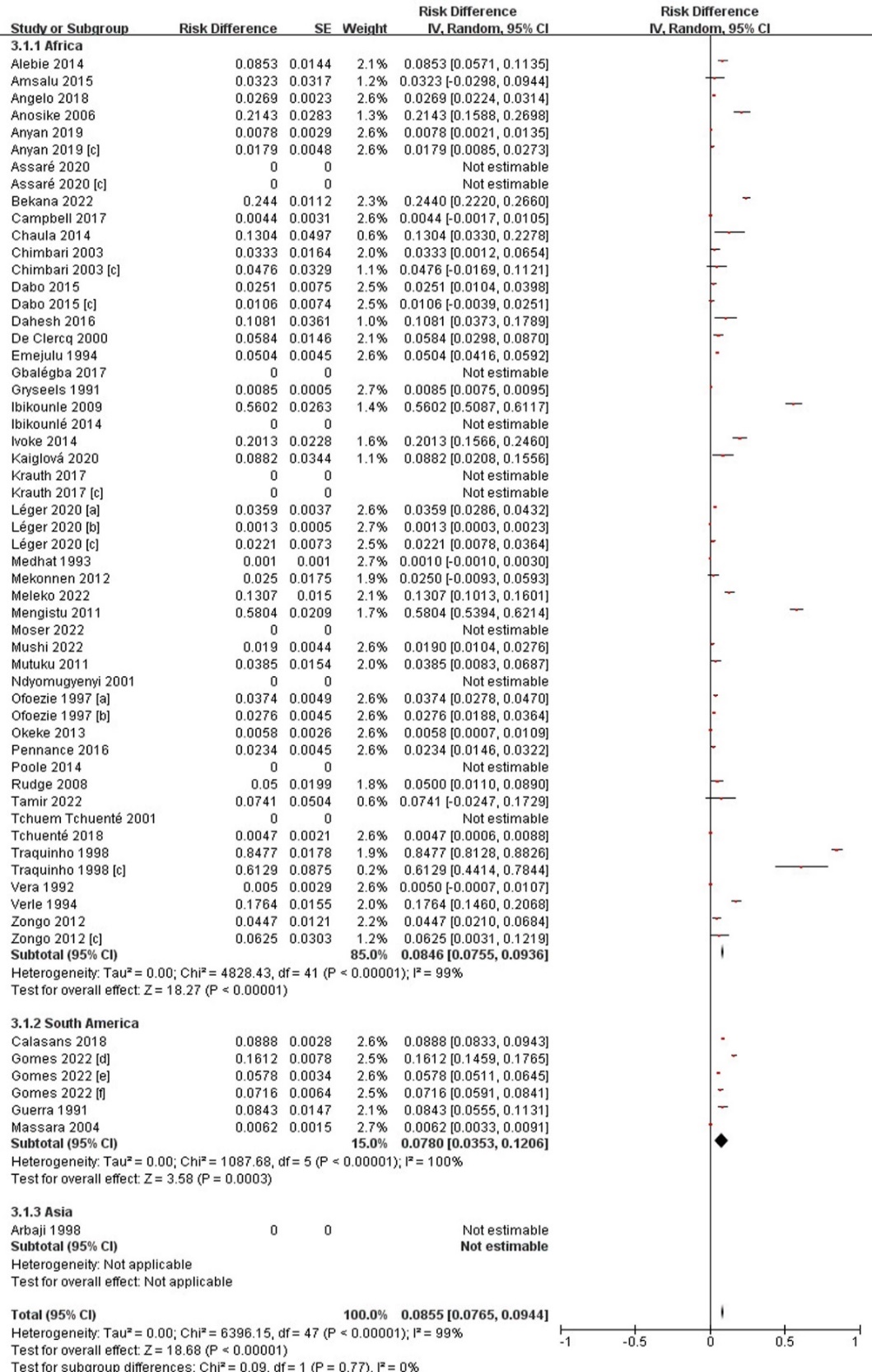


## Figure S7. Forest plot of subgroup PPE analysis of infectivity in the Africa, South America and Asia


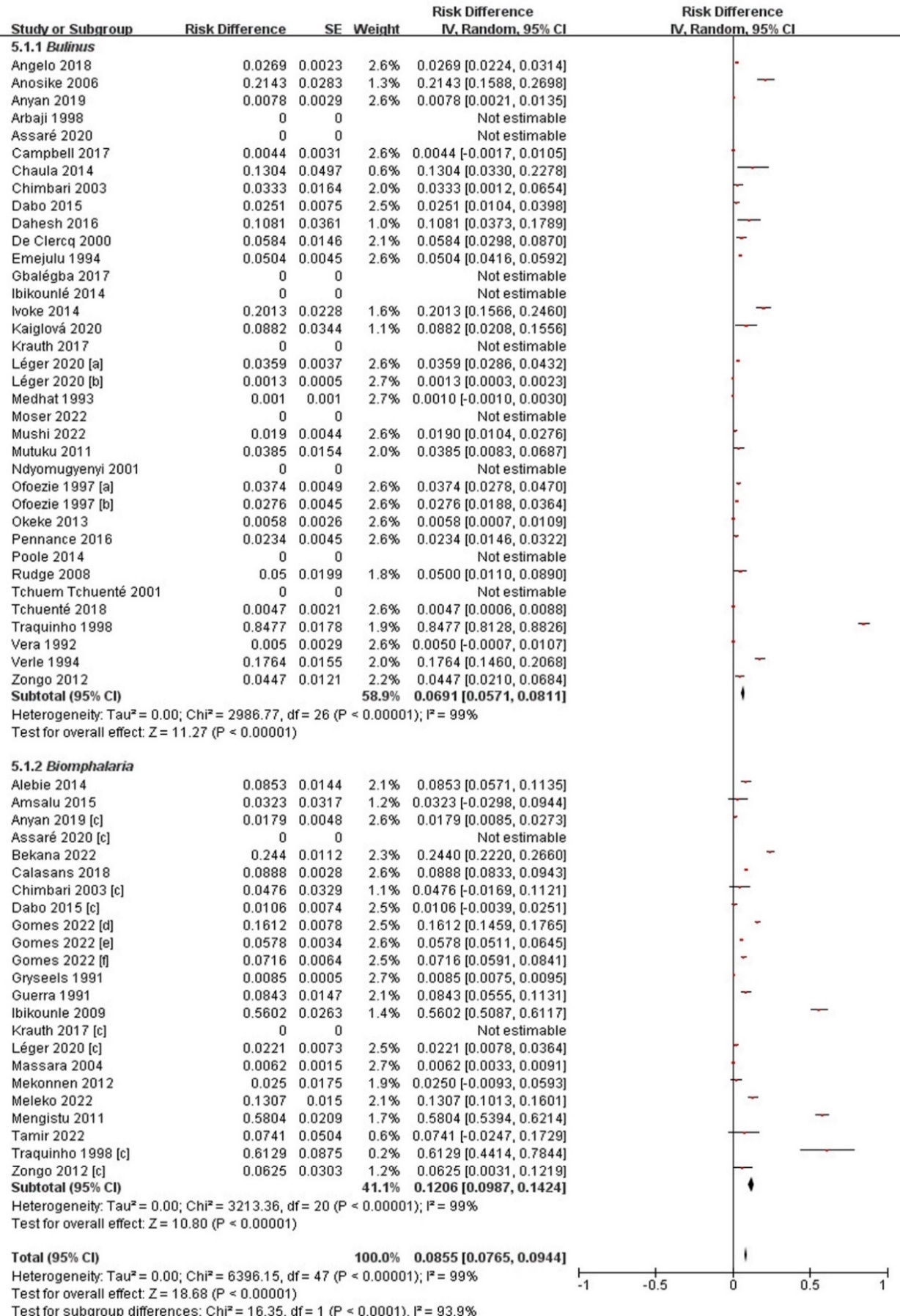


## Figure S8. Forest plot of subgroup PPE analysis of infection in the *Biomphalaria* spp. and *Bulinus* spp.

# Publication bias among intermediate host and definitive host studies


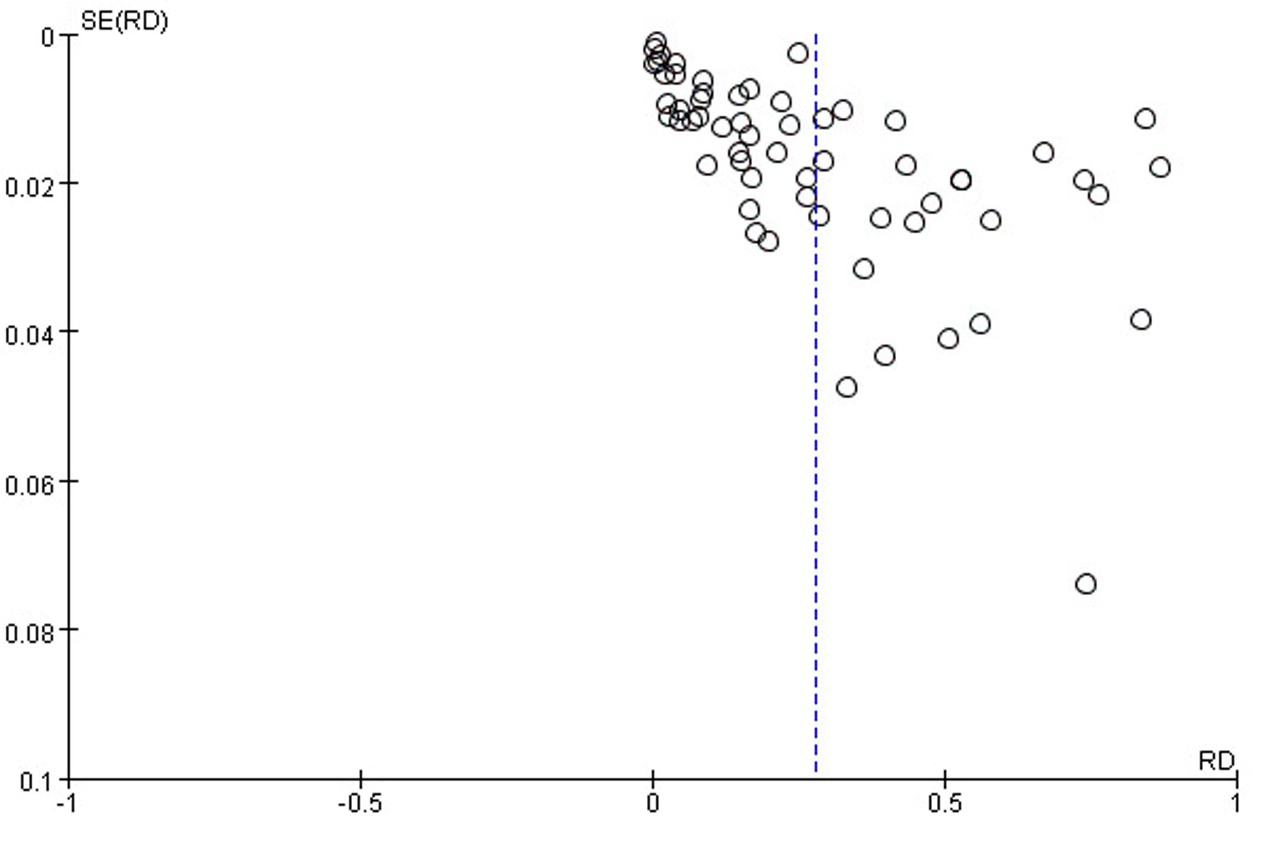


## Figure S9. Funnel plot with 95% confidence limit showing publication bias across studies on the prevalence of *S. mansoni* and *S. haematobium*


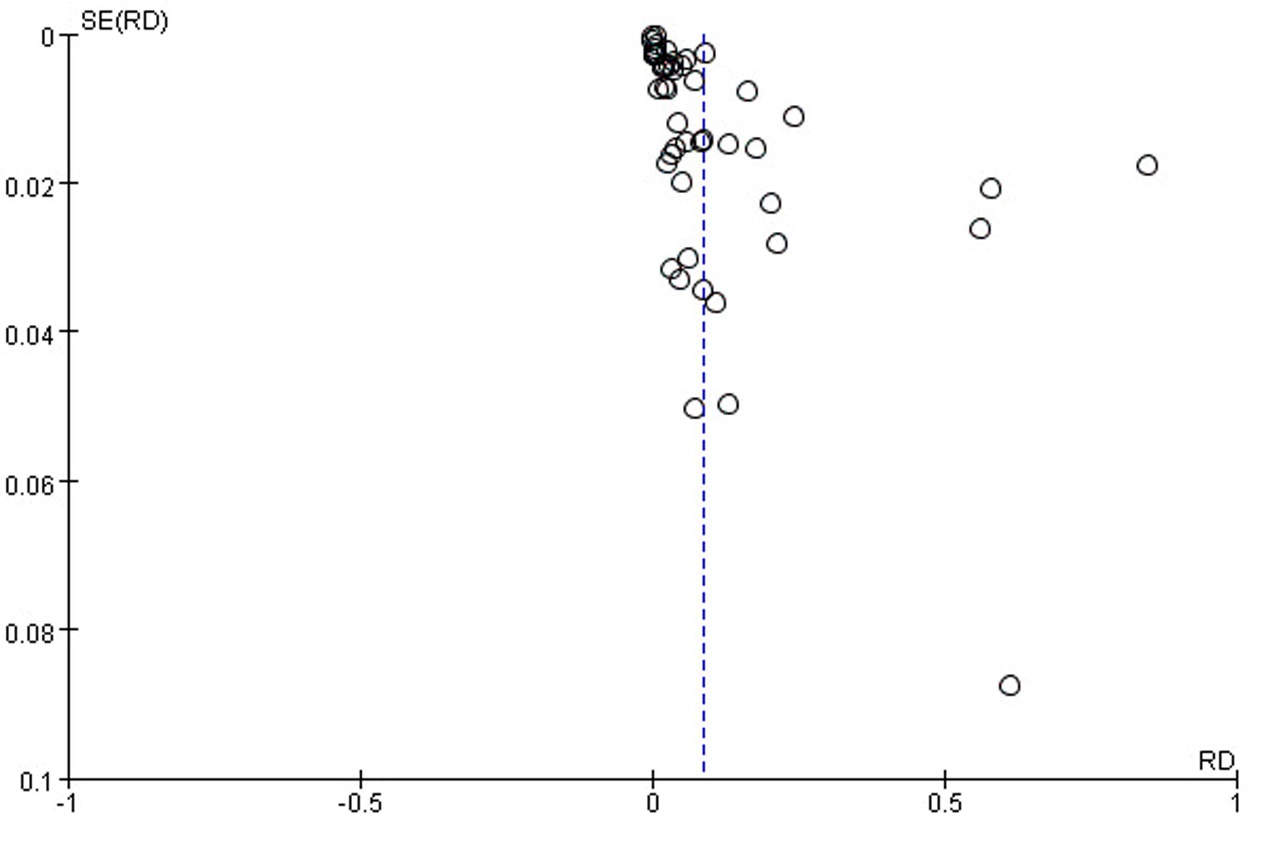


## Figure S10. Funnel plot with 95% confidence limit showing publication bias across studies on the prevalence of *S. mansoni* and *S. haematobium* among freshwater snails
